# Synergistic Effects of Alkali, Salt, and Thickness Reduction on the Preparation and Properties of Low‐Protein Noodles

**DOI:** 10.1002/fsn3.71870

**Published:** 2026-05-15

**Authors:** Ye Zi, Cuiping Shi, Zhenfeng Liu, Wei Cai, Jian Zhong

**Affiliations:** ^1^ Shanghai Key Laboratory of Pediatric Gastroenterology and Nutrition, Xinhua Hospital Shanghai Jiao Tong University School of Medicine Shanghai China; ^2^ Department of Clinical Nutrition, College of Health Science and Technology Shanghai Jiao Tong University School of Medicine Shanghai China; ^3^ Medical Food Laboratory Shanghai Institute for Pediatric Research Shanghai China; ^4^ Shanghai Pharma Qingchunbao‐Xinhua Hospital Precision Nutrition Research Center Chiatai Qingchunbao Pharmaceutical Group Limited Zhejiang Hangzhou China

**Keywords:** digestion characteristics, low‐protein foods, sensory evaluation, starch, texture

## Abstract

Low‐protein foods are important clinical diet requirements for some medical diseases. The work aimed to develop low‐protein noodles (LPNs) with good structural integrity, cooking and sensory properties. Based on a three‐step noodle fabrication process, the effects of low‐protein source, designated noodle width, drying condition, cooking time, alkali and salt addition, and dough sheet thickness on the properties of LPNs were explored to obtain ideal noodles. All results suggested these factors had obvious effects on the preparation of the LPNs. The optimal cooking times were 6 and 8 min for the wet and dry LPNs, respectively. Alkali and/or salt addition could increase the fastness to boiling to a certain extent, increase the sensory properties, and slow the starch digestion of the LPNs. Even without alkali and/or salt addition, dough sheet thickness reduction to 0.5 mm could significantly increase the fastness to boiling to an almost perfect extent. In addition, lower dough sheet thickness induced faster starch digestion percentages. The obtained LPNs were optimized with a low protein content of 0.62 g/100 g. The synergistic combination of the alkali of 0.5% low‐protein powder, the salt of 1.0% low‐protein powder, and a dough sheet thickness of ≤ 1.0 mm induced no gaps in the LPNs and almost no noodle fracture after cooking. Therefore, the synergistic use of alkali, salt, and thickness reduction could achieve the development of LPNs with good structural integrity. These results were beneficial to understand the relationship between the preparation and properties of the low‐protein foods.

## Graphical abstract

Based on a three‐step noodle fabrication process, the effects of low‐protein source, designated noodle width, drying condition, cooking time, alkali and salt addition, and dough sheet thickness on the properties of LPNs were explored to obtain ideal noodles. The synergistic use of alkali, salt, and thickness reduction could achieve the development of LPNs with good structural integrity.

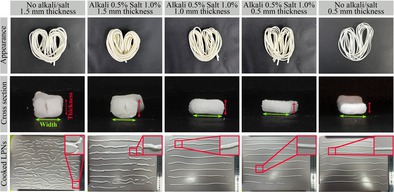

## 1 Introduction

Low‐protein foods (LPFs) are important daily and lifelong diet requirements for patients with diseases such as chronic kidney diseases, inherited urea cycle disorders, inherited organic acid metabolic disorders, and inherited amino acid metabolic disorders (Shi et al. 2025). A diet of the normal foods may induce excessive amounts of special amino acids in the blood, causing neurological damage (Faverzani et al. 2021). In contrast, a diet of LPFs may maintain low levels of the special amino acids in the blood, avoiding potential neurological damage. Therefore, the development and commercialization of LPFs are important clinical requirements for medical treatment.

Globally, many LPFs have been developed and commercialized until now. The number of commercialized LPFs varies in different countries (e.g., 73 in Portugal and 256 in Italy) (Pena et al. 2015). In addition, the developing countries generally have fewer LPFs than developed countries. In particular, the number of commercial LPFs is far less than the number of commercial regular foods. Moreover, some commercialized LPFs have relatively high protein contents, such as 2.5 g per 100 g of food (Pena et al. 2015). These commercialized LPFs have undesirable properties, such as high costs, unappealing organoleptic characteristics, nonideal palatability, and poor selections of cereal foods available to consumers. Proteins serve dual roles as nutrients and structural building blocks in foods. Particularly, protein (e.g., gluten in regular flour) plays a crucial role in determining the structural integrity and textural properties of baked foods (Ortolan and Steel 2017). Therefore, the development and application of LPFs have emerged as a significant research area in both food science and the medical field.

Over the past two decades, researchers have investigated how preparation factors affect the properties of LPFs to produce high‐quality foods. Low‐protein pasta formulations have been developed with low protein (2.94–5.76 g/100 g pasta) and phenylalanine (0.12–0.23 g/100 g pasta) contents (Yaseen and Shouk 2011). Low‐protein breads have been prepared with low protein (1.40–1.43 g/100 g dry sample) and phenylalanine (0.013–0.031 g/100 g bread) contents (Scortegagna et al. 2020). Low‐protein biscuits have been developed with low protein (e.g., 1.27 g/100 g biscuit) and phenylalanine (e.g., 0.05 g/100 g biscuit) contents (Azaripour and Abbasi 2020). However, the protein contents of these LPFs are still relatively high (≥ 1.27 g/100 g food). Previous work has suggested that proteins serve as important structural building blocks in foods (Foegeding 2015). Despite advances in the field, the formation mechanism of LPFs—particularly when proteins do not play a structural role—remains poorly understood. Consequently, future research should focus on both the creation of more LPFs and a deeper understanding of their fundamental formation mechanisms.

Noodles are a traditional staple food in many Asian countries. Three ingredients (flour, alkali, and salt) are common ingredients for regular noodle production (Li et al. 2018). The addition of inorganic salts (e.g., edible NaCl and Na_2_CO_3_) can improve the processing performance of the dough and the quality of the noodles (Obadi et al. 2022). Both edible salt and alkali can promote starch gelatinization in fresh noodles (doughs) during cooking by increasing starch viscosity (Li et al. 2018). Nevertheless, no formulation for low‐protein noodles (LPNs) has been reported to date. Therefore, it is of great interest to develop LPNs for the patients in Asian countries and to investigate the effects of ingredients and processing factors on their properties.

This work aimed to develop LPNs with good structural integrity, cooking, and sensory properties. The LPNs were made based on a three‐step (dough, wet noodle, and dry noodle preparation) process with three ingredients (low‐protein flour, alkali, and salt). First, SLPNs without alkali and salt addition were developed and evaluated. Second, the LPNs with alkali or salt addition were developed and evaluated. Third, the sheet thicknesses of the doughs were investigated. Finally, the in vitro digestion behaviors of the LPNs were analyzed. These results will provide valuable insights for the development of low‐protein foods.

## 2 Materials and Methods

### 2.1 Materials

Fengzheng high‐gluten flour (HGF, Weifang Fengzheng Flour Co. Ltd., Weifang City, Shandong Province, China). Aishushu low‐protein powder for noodles/dumpings (ALPPND) and Aishushu low‐protein powder for steamed buns (ALPPSB) (0.22 g and 0.47 g protein per 100 g powder, respectively) were purchased from Shanghai Zhunshen Food Dongtai Co. Ltd. (Dongtai City, Jiangsu Province, China). According to the ingredient lists, the ALPPND contains wheat starch, potato starch, acetate starch, corn starch, pea starch, acetylated monoglycerides and diglycerides, sodium alginate, guar gum, and vitamin C. According to the ingredient lists, the ALPPSB comprises wheat starch, potato starch, corn starch, acetate starch, pea starch, edible glucose, gluconic acid‐δ‐lactone, calcium carbonate, and sodium bicarbonate. Zhongen low‐protein flour for steamed buns (ZLPPSB, 0.32 g protein per 100 g powder) was purchased from Cangzhou Enji Biological Products Co. Ltd. (Cangzhou City, Hebei Province, China). According to the ingredient lists, the ZLPPSB contains wheat starch, pre gelatinized starch, oligofructose, oat fiber, pumpkin powder, and glucoamylase. Bakerdream edible alkali (sodium carbonate) was purchased from Angel Yeast Co. Ltd. (Yichang City, Hubei Province, China). Zhongyan refined edible salt was purchased from Zhongyan Shanghai Salt Industry Co. Ltd. (Shanghai, China). Cestbon Purified drinking water was purchased from China Resources Yibao Beverage (Yixing) Co. Ltd. (Yixing City, Jiangsu Province, China).

### 2.2 Preparation of LPNs

**FIGURE 1:**
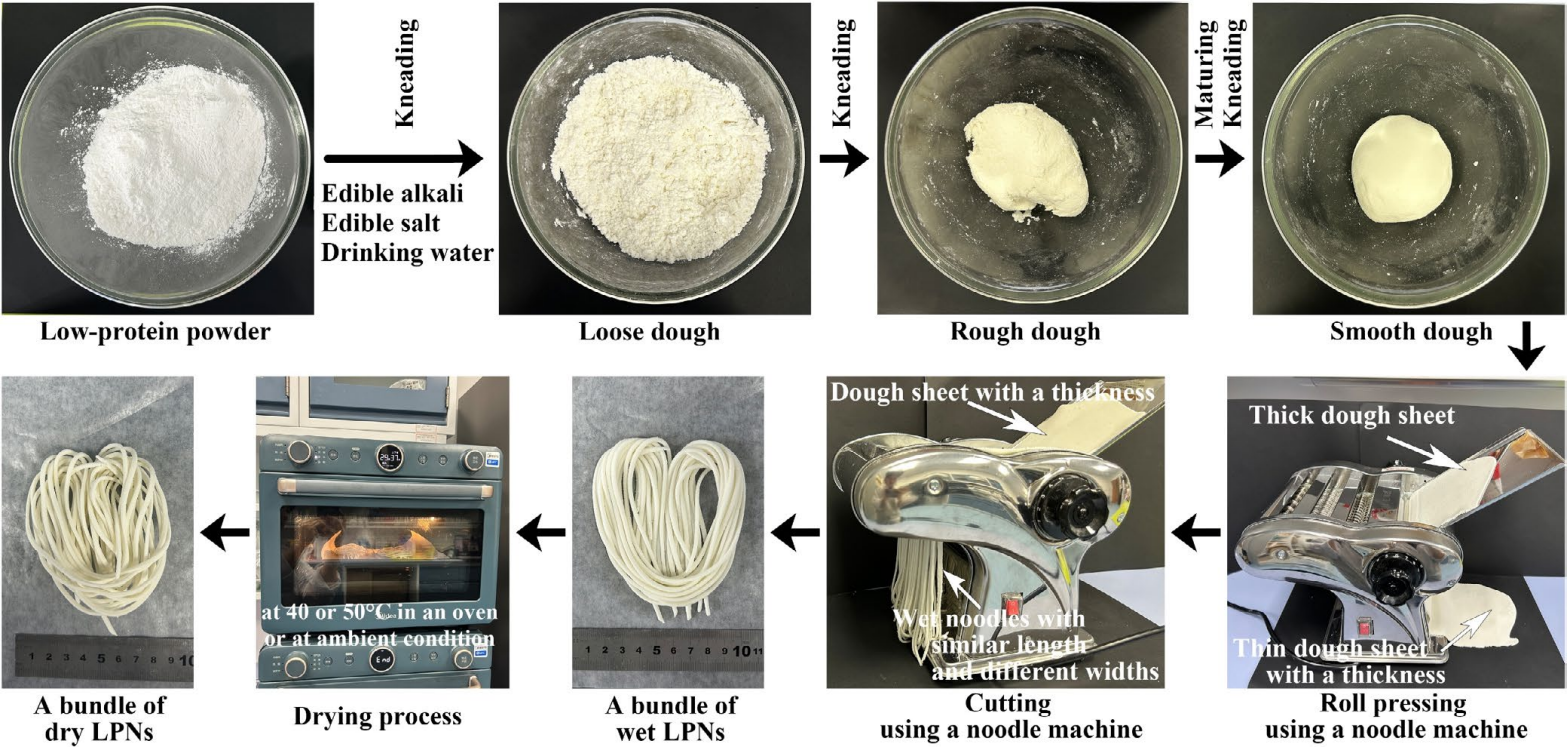
Preparation process of special low‐protein noodles (LPNs). Preparation of LPNs involved three steps: Smooth dough preparation (comprising loose dough preparation, rough dough preparation, and smooth dough preparation), wet noodle formation (consisting of roll pressing and cutting to form a bundle of wet LPNs), and noodle drying to form a bundle of dry LPNs. See details in the main text. The preparation conditions were as follows: Powder, Aishushu low‐protein powder for noodle/dumping (ALPPND); edible alkali additive amount, 0.50 g/100 g powder; edible salt additive amount, 1.00 g/100 g powder; designated width, 2.5 mm; designated thickness, 1.5 mm; and drying temperature, 50°C.

The preparation process of LPNs involved three steps (Figure 1): smooth dough preparation, wet noodle formation, and noodle drying (Yang et al. 2021). Briefly, ALPPND (100 g), edible alkali (0.0 or 0.5 g/100 g powder), edible salt (0.0 or 1.0 g/100 g powder), and drinking water (52 g/100 g powder) were mixed (According to the used raw materials, the obtained LPNs were named Control, Alkali 0.5%, Salt 1.0%, and Alkali 0.5% + Salt 1.0%). The mixtures were kneaded to form loose and then rough doughs in glass bowls. The rough doughs were sealed in the bowls with plastic wrap for 20 min to allow them to mature. Then, the doughs were kneaded to form smooth doughs.

The smooth doughs were pressed to form dough sheets with a designated thickness of 1.5 mm using a household electric noodle machine (BJM‐6, Deqing Baijie Electric Appliance Co. Ltd., Huzhou City, Zhejiang Province, China) with a roll pressing module. Then, the dough sheets were cut to form fresh wet noodles using the household electric noodle machine with the knife module to cut the noodles to similar designated lengths and a designated width of 2.5 mm.

The fresh wet noodles were bundled together. Then, the dry noodles were prepared by drying at 50°C in an electric oven (PT3520W‐G, Media). The dry noodles (about 3 g) were cut and ground into small pieces (size ≤ 2 mm) in a mortar. Then, they were placed in the sample cups with a volume of < 2/3, and the water activity values were measured using an AwTester Basic handheld water activity instrument device (Wisdom, Shanghai, China) (Zhang et al. 2024). The water activity values of commercial regular noodles were also measured and ranged from 0.5 to 0.6. Therefore, the drying processes were terminated when the water activity values were 0.5–0.6 (Drying time was 100–120 min). Finally, the dry noodles were sealed in PE/PE/PE/TIE/PA/TIE/PA seven‐layer composite grid‐embossed bags (Zhejiang Mingke Plastic Industry Co. Ltd., Taizhou City, Zhejiang Province, China) and stored at room temperature.

The effects of preparation factors were studied by varying one of the above parameters at a time. The following parameters were examined: powder (HGF, ALPPND, ALPPSB, and ZLPPSB), designated thickness (0.5, 1.0, 1.5, and 2.0 mm), designated width of (2.5, 4.0, and 9.0 mm), and drying condition (40°C in an electric oven for 120–150 min, 50°C in an electric oven for 100–120 min, or ambient room temperature condition for 8–9 h). These variations were used to prepare LPCs to analyze their effects on the properties of the final products.

### 2.3 Texture Properties

A TA.GEL texture analyzer (Shanghai BosinTech Co. Ltd., Shanghai, China) with a texture profile analysis (TPA) module and a TA/36R cylindrical probe was used to analyze the texture properties of doughs and boiled noodles (wet and dry noodles in Section 2.2) (Sun et al. 2019; Xu et al. 2023). The noodles were boiled in boiling water (500 mL) for 8 min and then drained for 3 min. Then, spherical doughs or three strips of boiled noodles (25 g) were placed on a flat plate (the “width” side was close to the flat plate). The samples were compressed to 50% of the original height at a speed of 1.00 mm/s. The pretest, test, and post‐test speeds were 3.00, 1.00, and 1.00 mm/s, respectively. The textural data were obtained using the commercial textural analyzer software.

### 2.4 Protein Contents

The protein contents of the LPNs and regular noodles were measured by Shanghai WEIPU Testing Technology Group Co. Ltd. (Shanghai, China). The procedures were performed according to the Chinese National Standards “GB 5009.5‐2016 Determination of protein in foods (Kjeldahl method).” Official reports were provided by the company and were qualified by both China Inspection Body and Laboratory Mandatory Approval (CMA) and China National Accreditation Service for Conformity Assessment (CNAS).

### 2.5 Cooking Time

The effect of cooking times on the LPNs was explored according to previous methods (Fu 2008; Xu et al. 2023) with slight modifications. Briefly, LPNs (5 g) were placed in boiling water (500 mL). At 2.0, 4.0, 6.0, and 8.0 min, one strip of LPN was taken out and cut into short 1.0‐cm LPNs. The cooking time was 8 min for other preparation factor analysis. The short LPNs were gently squeezed between two glass microslides and photographed using a digital camera.

### 2.6 Colorimetry

The color parameters (L*, lightness; a*, greenness‐redness; b*, blueness‐yellowness) of the dry SPLNs were analyzed using a colorimeter (DS‐220, Hangzhou CHNSpec Technology Co. Ltd., Hangzhou City, Zhejiang Province, China) (Erdem and Kaya 2021). Considering the sampling diameter of the colorimeter was 3.0 mm, the dry LPNs were obtained with a dough sheet thickness of 2.0 mm, a designated width of 5.0 cm, and a designated length of 5.0 cm. The color parameters were obtained by examining the samples using the colorimeter. The corresponding RGB values were obtained in Photoshop software by inputting the average color parameters (*L*, a*, and b*). Squares with the corresponding RGB values were inserted in the table in Microsoft Word and the square colors were set using the RGB values.

### 2.7 Cross Section Observation

The dry LPNs were broken, put on black paper, and photographed using a digital camera. The dry LPNs were broken off, inserted into a small dough, placed on a glass microslide, and observed using an ML53 optical microscope (Shanghai Minz, Shanghai, China) with a 5× objective (Zi et al. 2022).

### 2.8 Fracture Behaviors After Cooking

Three batches (eight strips for each) of LPNs were put into boiled water (500 mL). After 8 min, the LPNs were taken out and placed on a metal plate. The metal plate and a metal ruler were photographed using a digital camera. Then images were analyzed using Image J software (Version 2.1.0/1.53c, NIH, USA) with a set scale calibration to measure the length of the fractured LPNs (Zi et al. 2022). The numbers of the LPNs were summarized according to the length values and fitted with multipeak Gaussian in OriginPro 2021 (Version 9.8.0.200, OriginLab, USA) with a bin size of 2.0 cm (Martines et al. 2012).

### 2.9 pH in the Cooked Noodle

The pH in the cooked noodles was measured according to a previous work (Gao et al. 2023) with minor modifications. Briefly, ultrapure water (90 mL) was added to cooked noodles (10 g). The mixtures were homogenized at 8000 rpm for 1 min using a homogenizer (IKA T18, digital ULTRE TURRAX, Germany). Then, the pH of the mixtures was measured using a pH meter (FE28‐standard, Shanghai Mettler, China). The pHs of uncooked and cooked ultrapure water were also measured as controls.

### 2.10 Sensory Evaluation

Two bundles of dry LPNs were placed into boiled water (800 mL) in an electromagnetic cooker. After 8 min, the cooked LPNs were taken out and put into disposable paper bowls labeled with random digit numbers. A group of 18 panelists (20–35 years old) from the unit was recruited for the descriptive analysis of LPNs. The panelist number was close to that (20 panelists) of the descriptive analysis for regular noodles (Shao et al. 2019; Wang et al. 2022). The sensory evaluation did not require formal ethical approval because it did not involve medical research (Streule et al. 2024). The rights and privacy of all participants were safeguarded using a suitable protocol during the execution of the research (Pu et al. 2025).

All the participants were asked to sign informed consent forms to take part in this study. They were trained and screened by checking their responses to the sensory attributes: color, appearance, palatability, toughness, stickiness, smoothness, and taste. The sensory attributes and evaluation criteria followed a sensory evaluation process on regular noodle in a previous work (Wang et al. 2022). The panelists were asked to rate the noodles by seven sensory attributes, namely, “color (10 points),” “appearance (10 points),” “palatability (20 points),” “toughness (25 points),” “stickiness (25 points),” “smoothness (5 points),” and “taste (5 points).” The panel members were individually asked to rate the foods.

As for “color (brightness),” the evaluation criteria were: (i) bright color (8.5–10.0 point), (ii) weak bright color (6.0–8.4 point), and (iii) glossy brown color (0.0–5.9 point). As for “appearance (smooth and intact surface),” the evaluation criteria were: (i) intact and smooth appearance (8.5–10.0 point), (ii) middle level (6.0–8.4 point), and (iii) rough, expansive, or serious appearance (0.0–5.9 point). As for “palatability (the force to break noodles with the teeth)”, the evaluation criteria were: (i) appropriate strength (17.0–20.0 point), (ii) slightly hard or soft (12.0–16.9 point), and (iii) too hard or too soft (0.0–11.9 point). As for “toughness (biting strength and elasticity),” the evaluation criteria were: (i) appropriate biting strength and elasticity (21.0–25.0 point), (ii) middle level (15.0–20.9 point), and (iii) poor biting strength and elasticity (0.0–14.9 point). As for “Stickiness (strength of teeth sticking during chewing),” the evaluation criteria were: (i) refreshing and non‐sticking (21.0–25.0 point), (ii) middle level (15.0–20.9 point), and (iii) non‐refreshing and sticky (0.0–14.9 point). As for “Smoothness (smoothness during chewing)”, the evaluation criteria were: (i) smoothness (4.3–5.0 point), (ii) middle level (3.0–4.2 point), and (iii) poor smoothness (0.0–2.9 point). As for “Taste (taste during chewing),” the evaluation criteria were: (i) good taste (4.3–5.0 point), (ii) middle level (3.0–4.2 point), and (iii) uncomfortable taste (0.0–2.9 point). The total evaluation points were obtained by adding the seven sensory attributes together. The mean and standard deviation were calculated based on the obtained attribute scores from the panelists (*n* = 18) and statistical comparison was performed using one‐way ANOVA with Duncan test (*p* < 0.05).

### 2.11 In Vitro Starch Digestibility

The in vitro starch digestibility behaviors of the cooked dry LPNs were tested using a simulated intestinal model from a previous study (Guo et al. 2022) with slight changes. Starch digestion in the human body generally involves a sequential process that begins with α‐amylase and is followed by α‐glucosidase to produce glucose (Dhital et al. 2013). The temperature of the human body was simulated at 37°C (Shaha et al. 2021). The α‐amylase from porcine pancreas and glucoamylase had active pH ranges of 5.5–7.0 and 3.5–5.5, respectively (Wang et al. 2024). Therefore, the pH of the in vitro digestion system was set to 5.2. The concentrations of α‐amylase from porcine pancreas and glucoamylase in the in vitro digestion system were optimized to ensure starch hydrolysis of > 20%.

First, a mixed enzyme solution was prepared with α‐amylase and glucoamylase. The α‐amylase from porcine pancreas (129 mg, 9 U/mg, Shanghai Yuanye, China) was added to 10 mL of sodium acetate acetic acid buffer (0.2 mol/L, pH 5.2, Shanghai Macklin) and the mixture was magnetically stirred (300 rpm) for 30 min. Then, the mixture was centrifuged at 2039*× g* for 10 min using a laboratory centrifuge (5810 R, Eppendorf, Germany). The supernatant was mixed with 3 mL of glucoamylase solution (2000–3300 U/mL, Wanvi, Shanghai Linghan Scientific Instrument Co. Ltd., China) to form the mixed enzyme solution.

The dry LPNs (about 5 g) were placed in 500 mL of boiled water in an electromagnetic cooker. After 8 min, all the LPNs were taken out, drained, and cut into short 1.0‐mm pieces. Then, 200 mg ($W_{1}$) of LPN pieces (100 mg of the low‐protein powder was used as the control) were mixed with 5 mL of water. After adding seven 4.00 mm glass beads, the mixtures were boiled for 30 min. Then, the mixtures were mixed with 15 mL of sodium acetate acetic acid buffer. The total volume (V) was 20.0 mL. The mixtures were incubated at 37°C in a shaking bath (100 rpm, DKZ‐1, Shanghai Yiheng, China) for 10 min and a preheated (37°C for 10 min) mixed enzyme solution (0.1 mL) was then added. The mixtures were continuously shaken at 37°C for 180 min. At the designated time points (0, 20, 40, 60, 90, 120, and 180 min), a 0.2‐mL aliquot was mixed with 0.8 mL of absolute ethanol to stop the enzymatic reactions (dilution factor of 5). The mixtures were centrifuged (3889*× g*) for 5 min in an Eppendorf MiniSpin centrifuge (Hamburg, Germany).

The glucose concentrations (C, mg/mL) in the digestion solutions were measured using a D‐glucose (GOPOD) assay kit (Biosharp, Labgic Technology Co. Ltd., Shanghai, China) according to the following equation:

(1) $$C=C_{\text{Standard}}\times \frac{A_{\text{Sample}}-A_{\text{Water}}}{A_{\text{Standard}}-A_{\text{Water}}}\times D$$

where $C_{\text{Standard}}$ is the concentration (1 mg/mL) of the standard glucose solution, $A_{\text{Sample}}$ is the absorbance of the diluted sample solution, $A_{\text{Water}}$ is the absorbance of the ultrapure water, $A_{\text{Standard}}$ is the absorbance of the standard glucose solution, and D is the dilution factor of the sample during the treatment with the D‐glucose (GOPOD) assay kit.

The cooked dry LPNs were dried at 103°C for 18 h to remove moisture and obtain a constant weight of starch according to Chinese National Standard GB 5009.3–2016 (Determination of moisture content in foods) (Nie et al. 2022). The starch contents were calculated according to the following equation:

(2) $$\text{Starch content}\;\left(\%\right)=\frac{W_{4}-W_{2}}{W_{3}}\times 100$$

where $W_{2}$ is the weight of the glass bottle, $W_{3}$ is the weight of the cooked dry LPNs, and $W_{4}$ is the dried weight of the samples (the cooked dry LPNs and the glass bottle) after drying at 103°C.

Finally, the starch hydrolysis percentages were calculated according to the Equation (3):

(3) $$\text{Starch Hydrolysis}\;\left(\%\right)=\frac{\text{Hydrolyzed starch amount}}{\text{Starch amount in cooked}\;\mathrm{dry}\;\text{SLPNs}}=\frac{0.9\times C\times V}{W_{1}\times \text{Starch content}}\times 100$$

In this equation 0.9 is the factor that converts the glucose molecular mass into starch monomer units of anhydro‐glucose (the starch monomer unit) (Jiang et al. 2021).

The 1.5‐mm‐thick dry LPNs without alkali and salt addition were used as controls to analyze the effects of alkali and salt addition. Uncooked ALPPND low‐protein powder was used as a control to see the in vitro digestion of the low‐protein powder.

### 2.12 Statistical Analysis

For each experiment, three parallel samples were treated to obtain the data (presented as mean value ± standard deviation). All data were directly analyzed using one‐way ANOVA with Duncan test for statistical analysis (*p*‐value < 0.05) in IBM SPSS Statistics 26 software (Armonk, NY, USA). The assumptions of normality were not verified prior to ANOVA.

## 3 Results and Discussion

### 3.1 Effect of Low‐Protein Powder Source on Noodle Appearances and Textural Properties

**FIGURE 2:**
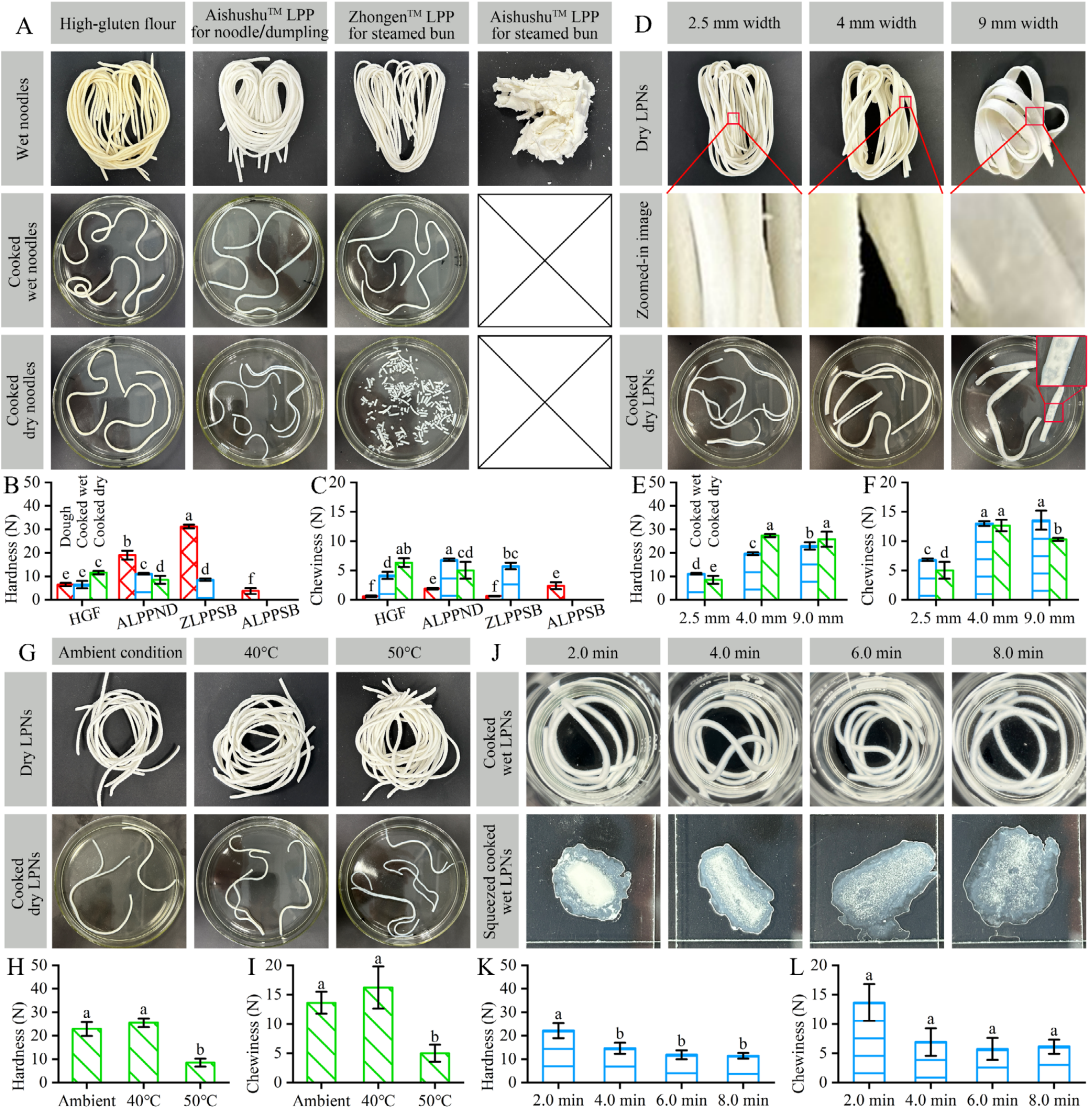
Cooking and textural properties of the LPNs without alkali and salt. The designated width and thickness were 2.5 and 1.5 mm, respectively, if not mentioned. The cooking time was 8 min if not mentioned. The drying condition was 50°C if not mentioned. The powder was ALPPND if not mentioned. (A) Wet, cooked wet, cooked dry LPNs with different flours: HGF, ALPPND, Aishushu low‐protein powders for steamed bun (ALPPSB), and Zhongen low‐protein powder for steamed bun (ZLPPSB). (B, C) Hardness and chewiness of the doughs, cooked wet noodles, cooked dry noodles with different powders. (D) Dry and cooked dry LPNs with different designated widths. (E, F) Hardness and chewiness of the cooked wet and cooked dry LPNs with different designated widths. (G) Dry and cooked dry LPNs with different cooking temperatures. (H, I) Hardness and chewiness of the cooked dry LPNs with different cooking temperatures. (J) Cooked wet and squeezed cooked wet LPNs with different cooking times. (K, L) Hardness and chewiness of the cooked wet LPNs with different cooking times. Different letters on the column indicate significant differences (*p* < 0.05) in each image.

LPNs were prepared using a three‐step baking method (dough preparation, noodle formation, and noodle drying), as shown in Figure 1. Four types of powders (one HGF and three low‐protein powders) were used to prepare the noodles. As shown in Figure 2A, wet noodles could not be easily prepared using ALPPSB, whereas wet LPNs were prepared using the other three powders. The yellow color of the wet noodles prepared using HGF resulted from the yellow color of the raw flour. All the three types of wet noodles were well‐cooked after 8 min in boiling water (named “Cooked wet noodles”).

All the three types of dry noodles showed different behaviors after cooking in the boiling water (named “Cooked dry noodles”). The cooked dry noodles from HGF had a good appearance. The cooked dry noodles made from ALPPND fractured along both the radial and axial directions. The cooked dry noodles made from ZLPPSB were short, measuring < 2 cm. It confirmed that proteins could serve important structural building blocks in foods (Foegeding 2015). Therefore, improving the structure of LPNs will help to obtain ideal LPNs with structural integrity. Furthermore, the protein contents of the HGF and ALPPND dry noodles were 9.26 and 0.62 g/100 g, respectively, as determined by Shanghai WEIPU Testing Technology Group Co. Ltd. (Shanghai, China). Therefore, ALPPND was chosen as the model low‐protein powder because its LPNs exhibited better behavior than those of the other two LPNs.

Four texture parameters (hardness, chewiness, springiness, and resilience) were used to analyze the textural properties of the LPNs. Hardness is related to the stiffness of the food and is determined by observing the maximum load reached during the first deformation cycle (Paredes et al. 2022). Chewiness is related to ease of biting and is obtained by multiplying the hardness by the cohesiveness and the springiness (Paredes et al. 2022). Springiness is related to the recovery of the food and is obtained by dividing the time required for the food to reach its maximum load by the time required for the first cycle (Paredes et al. 2022). Resilience is related to the plastic deformation of the food and is calculated by dividing the upstroke area by the downstroke area of the first compression cycle (Paredes et al. 2022).

The textural properties of cooked wet LPNs (Figure 2B,C, Figure S1), cooked dry LPNs (Figure 2B–C, Figure S1), and the doughs (Figure S2) made from different powders were analyzed. The raw materials affected the hardness (Figure 2B), chewiness (Figure 2C), springiness (Figure S1A), and resilience (Figure S1B) of the cooked wet and cooked dry noodles. All the cooked noodles showed different textural properties compared to the doughs (Figure S2). The cooked wet LPN made from ALPPND had higher hardness (Figure 2B), higher chewiness (Figure 2C), higher springiness (Figure S1A), and lower resilience (Figure S1B) than those prepared with HGF. The cooked wet LPN made from ZLPPSB had higher hardness (Figure 2B), higher chewiness (Figure 2C), higher springiness (Figure S1A), and similar resilience (Figure S1B) compared to those made from HGF. The cooked dry LPN made from ALPPND had lower hardness (Figure 2B), lower chewiness (Figure 2C), lower springiness (Figure S1A), and higher resilience (Figure S1B) than those prepared from HGF. Therefore, ALPPND was selected for developing LPNs.

Protein (e.g., gluten in regular flour) plays a crucial role in determining the structural integrity and textural properties of baked foods (Ortolan and Steel 2017). Therefore, appropriate foods additives are necessary to compensate for the absence of proteins in the low‐protein flours for the preparation of LPNs. The differing components of low‐protein powders may govern their performance in different food development. Previous work has suggested that sodium alginate and guar gum could improve noodle quality (Hong et al. 2021; Kaur et al. 2015). According to the ingredients lists of the three low‐protein powders, ALPPND contains sodium alginate and guar gum, whereas the other two powders (ALPPSB and ZLPPSB) don't. Sodium alginate is widely used as a thickener and stabilizer in food industry (EFSA ANS Panel (EFSA Panel on Food Additives and Nutrient Sources Added to Food) et al. 2017). Guar gum is also widely used, primarily as a thickener, in the food industry (EFSA ANS Panel (EFSA Panel on Food Additives and Nutrient Sources Added to Food) et al. 2017). Due to their functional properties, the sodium alginate and guar gum are important food additives to ensure the structural integrity of LPNs using low‐protein flours. Further studies are necessary to analyze the underlying molecular mechanism between these two food additives and starches to build the structural integrity and obtain good textural properties for the resulting LPNs.

### 3.2 Effect of Designated Noodle Width on Appearance and Textural Properties

The effect of the designated noodle widths (2.5, 4, and 9 mm) on the LPNs was studied, as shown in Figure 2D–F, Figures S3 and S4. Dry LPNs were obtained at these widths. However, some concavities were observed on the surfaces (the zoomed‐in images). Both the cooked wet and dry LPNs showed the presence of the concavities (Figure 2D: Cooked dry LPNs, zoomed‐in inset image). The cooked dry LPNs (Figure 2D) with the widths of 2.5 and 4 mm were fractured along both the radial and axial directions, whereas the cooked dry LPN with a width of 9 mm (Figure 2D) and the cooked wet LPNs of all thicknesses did not. The cooked 4‐ and 9‐mm thick dry (Figure 2D) and wet (Figure S3) LPNs exhibited concavities, which affected our sighting of the LPNs. It may result from the poor rheological properties of the ALPPND due to its low protein contents (Niu et al. 2017). Therefore, the noodle width of 2.5 mm was chosen to prepare the LPNs for the rest of this study. It was important to improve the fracture behaviors of the LPNs in both the radial and axial directions.

The textural properties of the cooked wet and dry LPNs with different widths are shown in Figure 2E–F, Figure S4. The 4‐ and 9‐mm‐wide cooked wet LPNs had higher hardness (Figure 2E) and chewiness (Figure 2F) than those with a width of 2.5 mm. The cooked wet LPNs showed similar springiness (Figure S4A) regardless of width. The resiliences of the 2.5‐ and 4‐mm‐wide cooked wet LPNs were similar (Figure S4B) and lower than that of the 9‐mm‐wide LPNs. In addition, the 4‐ and 9‐mm cooked dry LPNs had higher hardness (Figure 2E), chewiness (Figure 2F), springiness (Figure S4A), and resilience (Figure S4B) than the 2.5‐mm‐wide LPNs. These results confirmed that the noodle width affected the textural properties of cooked wet and dry LPNs. Moreover, wider noodles had higher textural properties (hardness, chewiness, springiness, and resilience).

### 3.3 Effect of Drying Condition on the Appearances and Textural Properties

The above studies also suggested that the drying step (cooked wet vs. dry LPNs) affected the appearance and textural properties of the LPNs before and after cooking. Therefore, it was necessary to analyze the effect of drying conditions on the LPNs. In this section, the LPNs were dried under three conditions: (i) Ambient condition at room temperature for 8–9 h; (ii) 40°C for 120–150 min; (iii) 50°C for 100–120 min. After drying, all the dry LPNs had good appearances without fractures (Figure 2G: Dry LPNs). However, all the cooked dry LPNs showed fracturing along both the radial and axial directions (Figure 2G: Cooked dry LPNs) compared with the dry LPNs (Figure 2G: Dry LPNs). Considering the drying time, the drying condition of 50°C for 100–120 min was chosen to prepare the LPNs in other studies.

The textural properties of the cooked dry LPNs with different drying conditions are shown in Figure 2H,I, Figure S5. Drying at the ambient condition (for 8–9 h) and 40°C (for 120–150 min) induced higher hardness (Figure 2H) and chewiness (Figure 2I), similar springiness (Figure S5A), and lower resilience (Figure S5B) compared to drying at 50°C (for 100–120 min). Previous work suggested the increased temperature (45°C for 12 min, 60°C for 8 min, 75°C for 6 min, 105°C for 200 s, 120°C for 150 s, 135°C for 105 s) could enhance the hardness and reduce the chewiness of common noodles (Li et al. 2016). The differences observed in this study might result from variations in the protein contents of the powder. Lower temperatures induced higher hardness and chewiness in low‐protein foods, but induced lower hardness and chewiness in regular foods.

### 3.4 Effect of Cooking Time on the Appearances and Textural Properties

The optimal cooking time was defined as the time when the hard core of the noodles disappeared (Xu et al. 2023). To analyze the effect of cooking time on the properties of the LPNs, noodles were cooked for 2‐min intervals (2.0, 4.0, 6.0, and 8.0 min). The resulting cooked wet (Figure 2J–L, Figure S6) and dry (Figure S7) LPNs were used to explore the effect of cooking time. The cooked wet LPNs (Figure 2J) showed no change in appearance compared with the wet LPNs. The hard cores of the LPNs were still present at cooking times of ≤ 4 min. However, no hard cores appeared for cooking times of ≥ 6 min (Figure 2J: Squeezed sample). Therefore, the cooking time of wet LPNs should be ≥ 6 min. For the dry LPNs, hard cores remained after cooking times of ≤ 6 min. However, no hard cores appeared with cooking times of ≥ 8 min (Figure S7: Squeezed sample). Therefore, the time of 8 min was chosen to cook the LPNs in other studies.

The textural properties (Figure 2K,L, Figure S6) of the cooked wet LPNs depended on the cooking time. In particular, a cooking time of 2 min induced higher hardness (Figure 2K), higher chewiness (Figure 2L), higher springiness (Figure S6A), and similar resilience (Figure S6B) compared to other cooking times (2–8 min). The hard cores of inadequately cooked foods induced higher hardness, chewiness, and springiness than those of the adequately cooked foods. Therefore, the inadequately cooked foods had different hardness, chewiness, and springiness from adequately cooked foods (Pematilleke et al. 2022).

### 3.5 Effects of Alkali or Salt Addition on the Appearances and Textural Properties

**FIGURE 3:**
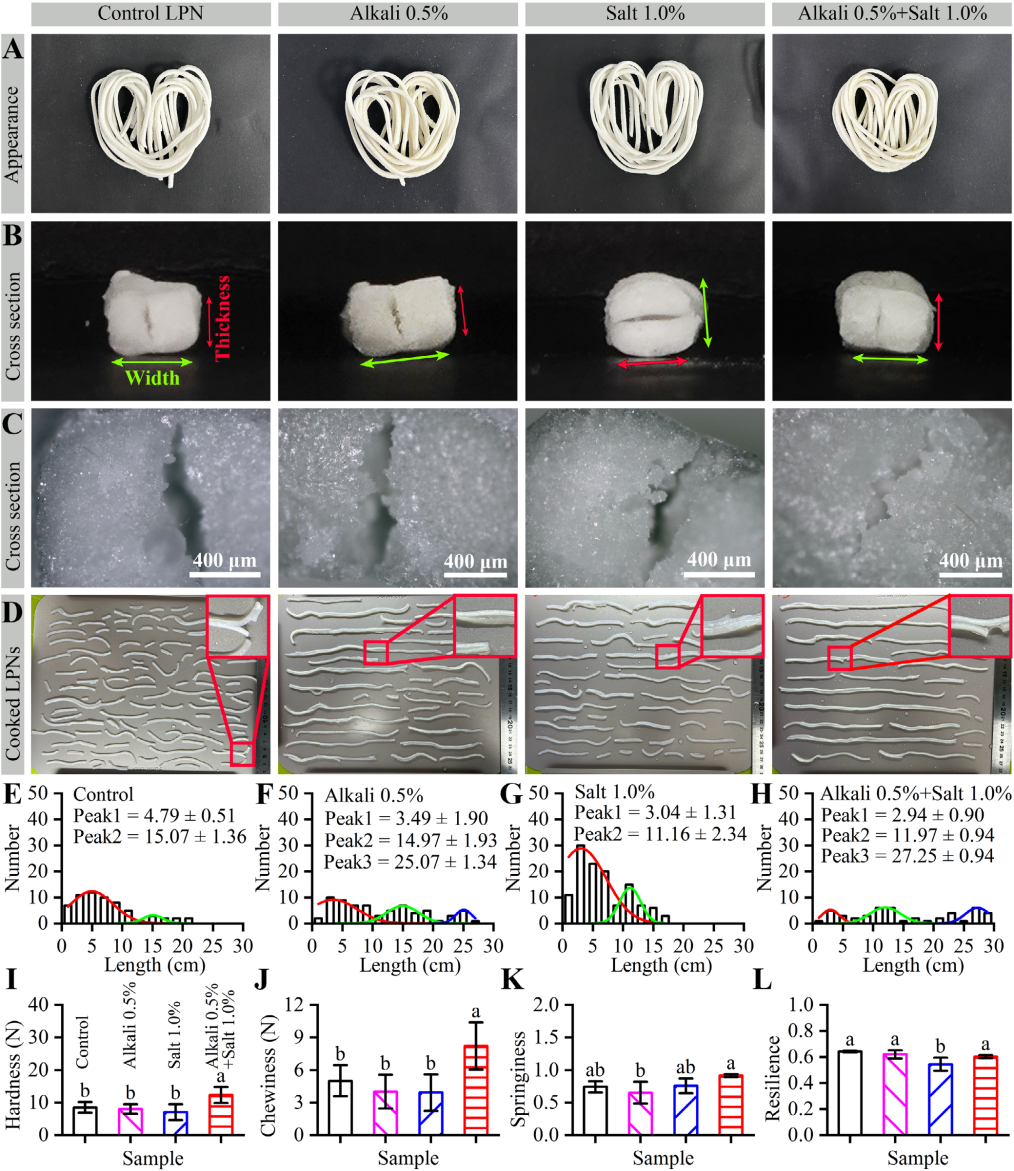
Effect of alkali and salt addition on the properties of the dry and cooked dry LPNs. The other preparation conditions were as follows: Powder, ALPPND; designated width, 2.5 mm; designated thickness, 1.5 mm; drying temperature, 50°C; cooking time, 8 min; edible alkali additive amount, 0.50 g/100 g powder; and edible salt additive amount, 1.00 g/100 g powder. The dry LPN without alkali and salt addition was used as a control and labeled as “Control LPN”. (A) Dry LPNs under a digital camera. (B) Cross section images of the dry LPNs using a digital camera. (C) Cross section images of the dry LPNs using an optical microscopy. Red and green arrows indicate the thickness and width directions of the dry LPNs. (D) Each batch of the cooked dry LPNs under a digital camera. The inset images show the enlarged regions. (E–H) LPN numbers versus the lengths of the cooked dry LPNs. The Peak 1, 2, and 3 values show the fitted values via multiple Gaussian peak fitting. (I–L) Textural properties of cooked dry LPNs. Data in (I–L) are presented as mean ± standard deviation (error bars). Different letters on the column indicate significant differences (*p* < 0.05) in each image.

The LPNs were prepared with alkali (0.5% of low‐protein powder), salt (1.0% of low‐protein powder), and a combination of alkali and salt (0.5% and 1.0%, respectively). The resulting dry LPNs had good appearances (Figure 3A). Moreover, the samples with added alkali were a light yellow color, whereas the other samples were white. The colorimetry results (Table 1) showed that the alkali addition decreased L* and a*, but increased b* for the dry LPNs. These results were consistent with the effect of added alkali amounts on common wheat noodles (Xu et al. 2022). Alkali addition increased the pH of the doughs and noodles, and the internal colorless flavones (apigenin glycosides) became yellow in the alkaline environment (Xu et al. 2022). In addition, salt addition increased L*, decreased a*, and increased b* of the dry LPNs. It suggested that salt did significantly change the color of the LPNs, which was also consistent with the appearance of LPNs (Figure 3A and Table 1: Color square).

**TABLE 1:** Color parameters of the dry LPNs with edible alkali and/or salt additions.

Sample	L*	a*	b*	Color square
Control	90.34 ± 0.42^b^	0.63 ± 0.12^a^	‐3 ± 0.31^d^	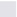
Alkali 0.5%	86.07 ± 0.61^d^	−1.88 ± 0.21^c^	11.78 ± 0.79^a^	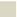
Salt 1.0%	93.33 ± 0.84^a^	−0.41 ± 0.14^b^	3.46 ± 0.43^c^	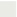
Alkali 0.5% + Salt 1.0%	88.05 ± 0.64^c^	−1.68 ± 0.1^c^	9.88 ± 0.77^b^	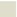

*Note:* The samples were the same as those in Figure 3. Values are expressed by mean ± standard deviation and the different letters in the same column indicate significant differences (*n* = 18, *p* < 0.05).

The cross‐sectional images of these dry LPNs were studied to analyze the effects of alkali and salt addition on the preparation of LPNs. As shown in Figure 3B, the cross sections of the control LPNs (no alkali and salt addition) showed gaps along the thickness direction. These gaps might be formed during the cutting step (Figure 1). They might result from the low rheological properties of the ALPPND due to low protein content (Niu et al. 2017). Neither alkali nor salt addition could decrease the gap sizes in the cross section of the LPNs; however, the synergistic use of alkali and salt addition could decrease the gap sizes. The optical microscopy images (Figure 3C) also confirmed the digital camera results (Figure 3B). Moreover, the optical microscopy images (Figure 3C) showed that the cross sections were relatively uniform aside from the gaps at the sub‐micrometer scale.

The dry LPNs were cooked for 8 min, and their appearances and textural properties were analyzed, as shown in Figure 3D–L. In Figure 3D, the control LPNs consist of short linear noodles, with lengths significantly shorter than the original noodle lengths (25–30 cm). The Gaussian fitting of number versus length (Figure 3E) suggested that the control LPNs were mainly 4.79 ± 0.51 and 15.07 ± 1.36 cm long. These results suggested that the control LPNs fractured along the axis direction. Both alkali and salt addition could increase the LPN lengths (Figure 3F,G vs. Figure 3E). Moreover, alkali addition (Figure 3F) had a better ability of LPN length protection than salt addition (Figure 3G). In addition, the synergistic use of alkali and salt addition could further increase the LPN lengths (Figure 3H vs. Figure 3E). The salt addition could improve the dough's processing performance and the regular noodle's quality (Obadi et al. 2022). Both edible salt and alkali could promote starch gelatinization in fresh noodles (doughs) during cooking by increasing starch viscosity (Li et al. 2018), which might increase the robustness of the LPNs to boiling (Figure 3D–H). However, the fractural behaviors of the cooked dry LPNs were present along the axis direction (Figure 3D: Enlarged inset images), which was consistent with the gaps observed in the dry LPNs (Figure 3B). Moreover, the addition of alkali or salt addition did not improve the fractural behaviors of the cooked dry LPNs (Figure 3D: Enlarged inset images).

The textural properties of the cooked dry LPNs are shown in Figure 3I–L. Alkali addition had no obvious effects on the hardness (Figure 3I), chewiness (Figure 3J), springiness (Figure 3K), and resilience (Figure 3L) of the cooked dry LPNs. Salt addition did not affect the hardness (Figure 3I), chewiness (Figure 3J), and springiness (Figure 3K) of the cooked dry LPNs, but did decrease the resilience (Figure 3L) of the cooked dry LPNs. The addition of alkali to dough normally leads to increased viscosity, strengthened gluten networks (resulting from enhanced protein polymerization/cross‐linking), and improved elasticity, all of which collectively affect dough hardness (Xu et al. 2022). The synergistic use of alkali and salt addition significantly increased the hardness (Figure 3I) and chewiness (Figure 3J) of the cooked dry LPNs, but had no obvious effects on their springiness (Figure 3K) and resilience (Figure 3L). Therefore, the hardness and chewiness were the key textural properties of the cooked dry LPNs.

The pH of four cooked noodles was measured to analyze the effects of alkali or salt addition on the noodles. The pHs of the uncooked and cooked ultrapure water were 7.08 ± 0.05 and 7.05 ± 0.00, respectively. It suggested that the cooking process had no effects on the ultrapure water. Further, four cooked LPNs containing alkali (0.5% of low‐protein powder), salt (1.0% of low‐protein powder), alkali and salt (0.5% and 1.0%, respectively), and without alkali/salt (Control LPN) had the pH of 9.75 ± 0.18, 8.59 ± 0.04, 10.09 ± 0, and 8.37 ± 0.09, respectively. Thus, refined salt slightly and alkali significantly increased the pH of LPNs. They were consistent with previous reports that alkali increased the pH of foods (Song et al. 2024).

These results suggested edible salt and alkali increased the robustness of the LPNs to boiling with different degrees (Figure 3D–H). Both edible salt and alkali could promote starch gelatinization in fresh noodles (doughs) during cooking by increasing starch viscosity (Li et al. 2018), which explained that they both could increase the robustness of the LPNs to boiling. The addition of alkali could improve the disulfide bond linkage between globulin and gluten in wheat noodle dough, significantly enhancing its processing properties (Zhang et al. 2022). Additionally, salt could induce non‐covalent interactions between globulin and gluten in wheat noodle doughs, slightly improving its processing properties (Zhang et al. 2022). Therefore, alkali addition (Figure 3F) increased the lengths of the cooked LPN more than salt addition (Figure 3G). Moreover, the synergistic use of alkali and salt addition could significantly decrease the gaps in the cross section (Figure 3B,C) and increase the noodle length (Figure 3D–H), which in turn increased the hardness (Figure 3I) and chewiness (Figure 3J) of the LPNs.

### 3.6 Effects of Alkali or Salt Addition on Sensory Properties

The sensory properties of the LPNs were evaluated in order to analyze the effects of alkali or salt addition on the LPNs (Table 2). Both alkali and salt additions increased the six sensory attributes (appearance, palatability, toughness, stickiness, smoothness, and taste). It was consistent with previous reports that alkaline conditions positively affected the color and flavor of foods (Deleu et al. 2019). Additionally, salt has been shown to contribute to the taste and other sensory characteristics of a food by increasing the volatility of aroma compounds and mouth feeling (Hoppu et al. 2017). Alkali addition decreased, while salt addition increased the color attribute. Therefore, both the alkali and salt addition increased the total sensory scores of the LPNs. Moreover, the synergistic use of alkali and salt addition increased the six sensory attributes (appearance, palatability, toughness, stickiness, smoothness, and taste), whereas they decreased the color attribute. Therefore, their synergistic use increased the total sensory scores of the LPNs.

**TABLE 2:** Sensory score results of noodles at different edible salt and edible alkali additions.

Sample	Color (10)	Appearance (10)	Palatability (20)	Toughness (25)	Stickiness (25)	Smoothness (5)	Taste (5)	Sum (100)
Control	8.41 ± 1.08^ab^	7.86 ± 1.22^a^	16.33 ± 1.71^a^	19.33 ± 3.31^b^	21.58 ± 2.33^a^	4.18 ± 0.62^a^	4.28 ± 0.68^a^	81.97 ± 7.33^b^
Alkali 0.5%	7.97 ± 1.01^b^	8.47 ± 1.11^a^	16.67 ± 2.50^a^	20.83 ± 2.46^ab^	21.81 ± 2.40^a^	4.20 ± 0.60^a^	4.39 ± 0.46^a^	84.33 ± 7.76^ab^
Salt 1.0%	8.96 ± 0.73^a^	8.26 ± 0.82^a^	16.61 ± 1.29^a^	19.50 ± 2.79^b^	21.67 ± 2.19^a^	4.22 ± 0.60^a^	4.44 ± 0.49^a^	83.65 ± 5.17^ab^
Alkali 0.5% + Salt 1.0%	7.93 ± 1.11^b^	8.50 ± 1.07^a^	17.64 ± 1.85^a^	22.31 ± 1.66^a^	22.28 ± 2.14^a^	4.36 ± 0.39^a^	4.42 ± 0.49^a^	87.42 ± 6.18^a^

*Note:* The samples were the same as those in Figure 3. Values are expressed by means ± standard deviation, and the different letters in the same column indicate significant differences (*n* = 18, *p* < 0.05).

The effects of the alkali and salt addition on the palatability, toughness, and stickiness differed from their effects on the textural properties (Figure 3I–L). These sensory attributes were affected by many factors. Previous work has suggested that adding 0% to 3% salt could improve the sensory properties of the noodles by enhancing their protein network structure in the noodles (Obadi et al. 2022). Furthermore, adding alkali (0.1%–0.2% dosage) could enhance the sensory properties of common noodles by promoting the formation of a protein gluten network (Xu et al. 2022). In this work, the alkali or salt addition might enhance the protein network in the LPNs. Therefore, the addition of alkali (0.5% powder) and salt (1.0% powder) could improve the sensory properties of the LPNs.

Among the seven sensory attributes, only color showed a behavior different from the total sensory score. Alkali decreased the color attributes, whereas salt increased them. They were consistent with the L* in the colorimetry results (Table 1), as discussed above. The sensory evaluation of the color was consistent with the lightness of the LPNs. Further development of the LPNs should consider increasing their lightness of the LPNs.

### 3.7 Effect of Dough Sheet Thickness on the Appearance and Textural Properties

**FIGURE 4:**
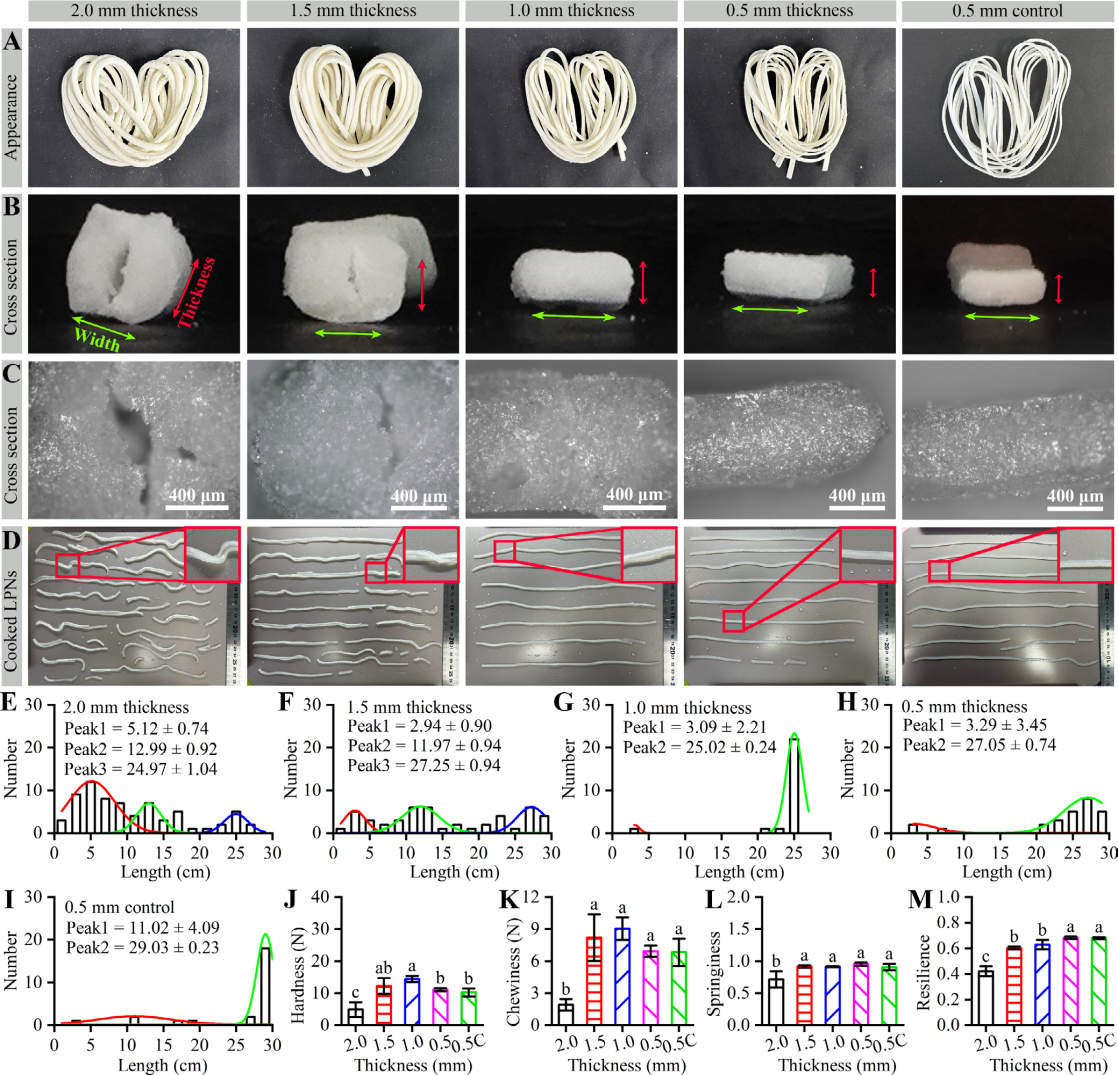
Effect of dough sheet thicknesses on the properties of dry and cooked dry LPNs. The alkali and salt additive amounts were 0.5% and 1.0%, respectively, of low‐protein powder. The other preparation conditions were as follows: Powder, ALPPND; designated width, 2.5 mm; and drying temperature, 50°C. Dry LPNs with a thickness of 0.5 mm and without alkali and salt addition was used as a control and labeled as “0.5C”. (A) Dry LPNs under a digital camera. (B) Cross section images of the dry LPNs using a digital camera. Red and green arrows indicate the thickness and width directions of the dry LPNs. (C) Cross‐sectional images of dry LPNs using an optical microscopy. (D) Each batch of the cooked dry LPNs under a digital camera. The inset images show the enlarged regions. (E–I) LPN numbers versus lengths of the cooked dry LPNs. The Peak 1, 2, and 3 values show the fitted values via multiple Gaussian peak fitting. (J–M) Textural properties of the cooked dry LPNs. Data in (J–M) are presented as mean ± standard deviation (error bars). Different letters on the column indicate significant differences (*p* < 0.05) in each image.

The LPNs were prepared with different thicknesses (2.0, 1.5, 1.0, and 0.5 mm) with 0.5% alkali and 1.0% salt to analyze the effects of the dough sheet thickness on the appearance and textural properties of LPNs. The dry LPN with a thickness of 0.5 mm without alkali and salt was used as a control (labeled with “0.5C” in Figure 4). The obtained LPNs showed good appearance and similar colors (Figure 4A). It was reasonable that differences in thickness did not affect the colors of the LPNs.

The cross‐sectional images of these samples were examined to investigate the impact of dough sheet thickness on the LPNs. As shown in Figure 4B,C, the gap sizes decreased with decreasing dough sheet thickness. Moreover, no gaps appeared for dough sheet thicknesses of ≤ 1.0 mm. It is interesting that no gaps appeared at the cross‐sectional image of the control LPN. Therefore, the thickness reduction was adequate to eliminate the gaps. In addition, the optical microscopy images (Figure 4C) showed that the cross sections were relatively uniform, aside from the sub‐micrometer‐scale gaps.

The dry LPNs were cooked for 8 min, and the appearances and textural properties were analyzed, as shown in Figure 4D–M. As shown in Figure 4D–I, the dough thickness of ≤ 1.0 mm significantly decreased the number of short LPNs compared to the thickness over 1.5 mm. Moreover, at a dough sheet thickness of 0.5 mm, only a few of the LPNs fractured along the axial direction (Figure 4D: Enlarged inset images). Therefore, reduced noodle thickness significantly increased the cooking ability of the LPNs, and 0.5 mm was the best dough sheet thickness to prepare LPNs.

The textural properties of the cooked dry LPNs are shown in Figure 4J–M. Dough sheet thickness of ≤ 1.5 mm induced higher textural properties than a sheet thickness of 2.0 mm, mainly due to the presence of gaps in the LPNs (Figure 4B,C). Previous work has suggested that the too thick and too thin noodles were not suitable for eating; a moderate thickness (2 mm) was the best choice for wheat flour noodles (Wang et al. 2020). However, our results suggested that small thicknesses (≤ 1.0 mm) were appropriate for the preparation of LPNs. The differences might be due to the different protein contents in the raw powders. Proteins are important structural building blocks in foods (Foegeding 2015). Therefore, a small thickness was required for the preparation of LPNs due to the low protein content of the raw powder.

According to the above analysis, dry LPNs could be prepared with different dough sheet thicknesses (Figure 4A). Reducing the dough sheet thickness could significantly decrease the gaps in the cross section (Figure 4B,C) and increase the noodle length (Figure 4D–I). These changes increased the robustness to boiling (Figure 4D–H) and textural properties (Figure 4J–M) of the LPNs. Moreover, a dough sheet thickness of 0.5 mm completely inhibited the gaps (Figure 4B–C) and improved the robustness to boiling (Figure 4D–H) of the LPNs even without alkali and salt addition.

### 3.8 In Vitro Digestion Behaviors

**FIGURE 5:**
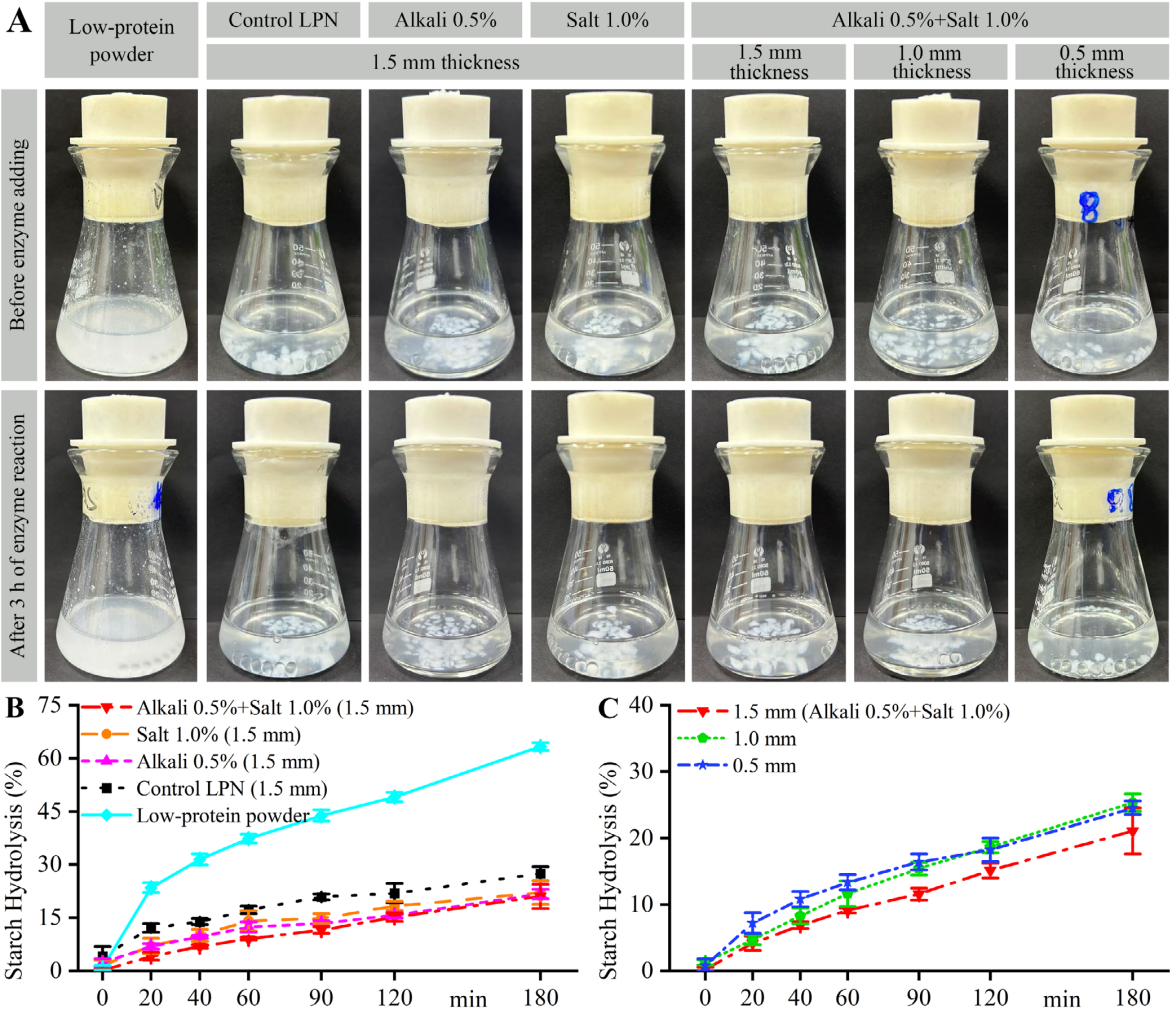
In vitro digestion behaviors of the cooked dry LPNs. The other preparation conditions were as follows: Powder, ALPPND; designated width, 2.5 mm; drying temperature, 50°C; and cooking time, 8 min. The dry LPN with a thickness of 1.5 mm and without alkali and salt addition was used as a control and labeled as “Control LPN (1.5 mm)”. The uncooked ALPPND low‐protein powder was used as control and labeled as “Low‐protein powder”. (A) Appearances of the samples in the digestion fluids. (B) Starch hydrolysis of cooked dry LPNs with different alkali/salt additive amounts. The designated thickness was 1.5 mm. (C) Starch hydrolysis of cooked dry LPNs with different designated dough sheet thicknesses. The alkali and salt additive amounts were 0.5% and 1.0% of low‐protein powder, respectively.

The in vitro digestion behaviors of cooked dry LPNs with varying alkali or salt addition and different dough sheet thicknesses, as well as dough sheet thicknesses, were analyzed using a simulated intestinal model (Guo et al. 2022). As shown in Figure 5A, the LPN pieces were present before the addition of mixed enzyme (α‐amylase+glucoamylase) and after 3 h enzyme reaction. All of the cooked dry LPNs were cut into short 1‐mm pieces, and the digestion process did not significantly change their morphologies.

The starch digestion curves of the cooked dry LPNs with different amounts of added alkali or salt are shown in Figure 5B. The starch hydrolysis rate of low‐protein powder increased rapidly within 0–20 min and then slowly within 20–180 min, a typical logarithmic growth curve. This trend was consistent with the starch digestion of whole buckwheat noodles (Xu et al. 2023). All the LPNs had slower digestion rates (Figure 5B) than the low‐protein powder (Figure 5B), possibly because the LPNs had significantly lower surface‐to‐volume ratios than the low‐protein powder. Moreover, the alkali or salt addition slowed the starch digestion rates. Previous work has suggested that alkali treatment could increase the digestibility of starches by removing the proteins and lipids (mostly from the surface) from the starch granules (Wang et al. 2014). The LPNs had low protein and lipid contents according to the ingredient list of the raw materials. Therefore, the digestion of starch generally did not require removing the proteins and lipids from the starch granules. The active pH ranges of α‐amylase from porcine pancreas and glucoamylase were 5.5–7.0 and 3.5–5.5, respectively (Wang et al. 2024). Therefore, 5.2 was used as the pH of the in vitro digestion system. The alkali (sodium carbonate) might inhibit the activity of glucoamylase, thereby decreasing the starch hydrolysis rates. NaCl could significantly decrease the activity of α‐amylase (Liu et al. 2018), thereby decreasing the starch hydrolysis rates.

The starch digestion curves of the cooked dry LPNs with different dough sheet thicknesses are shown in Figure 5C. All of the starch hydrolysis percentages increased with increasing digestion time. During the initial 0–90 min period, the thinner LPNs had higher starch hydrolysis percentages, indicating that starch hydrolysis percentages decreased with increasing LPN sizes. It could be explained that the smaller surface area induced lower enzyme digestion rates (Dhital et al. 2010). Between 90 and 180 min, the 1.0‐mm‐thick LPN showed similar starch hydrolysis percentages to that with a thickness of 0.5 mm, and they showed higher starch hydrolysis percentages than the 1.5‐mm‐thick LPN.

## 4 Conclusion

In summary, this study explored the synergistic effects of alkali, salt, and thickness reduction on the preparation, appearance, texture, sensory properties, and in vitro digestibility of salted LPNs. The results demonstrated that the combination of these three factors was effective in producing LPNs. Specifically, the synergistic use of 0.5% alkali (based on low‐protein powder), 1.0% salt, and a dough sheet thickness of ≤ 1.0 mm resulted in noodles with no visible gaps in the cross‐section and almost no fracture after cooking. This work provides valuable insights into the relationship between processing parameters and the properties of LPNs.

Owing to their low protein content, LPNs represent an excellent low‐protein food option, supplying energy while helping to reduce the risk of neurological damage associated with the intake of certain amino acids. They allow patients to enjoy noodle‐based dishes as part of a normal diet. These noodles can be consumed alongside other protein‐based products, such as phenylalanine‐free medical foods, to meet total nutritional requirements (e.g., in patients with phenylketonuria). Further exploration is required to confirm their nutritional function for the diet management of the patients.

The main limitation of this study lies in the irregularity and lack of precision in noodle shaping, which may have compromised the accuracy of the results, particularly those related to textural properties. Variations in noodle dimensions and geometry could introduce inconsistencies during mechanical testing, thereby affecting the reliability of the textural data. Another limitation concerns the number of panelists involved in the sensory evaluation. A relatively small panel size may reduce the statistical power of the sensory analysis and limit the generalizability of the findings. Future studies should consider recruiting a larger and more diverse panel to enhance the robustness and representativeness of the sensory assessments.

Additionally, further research is needed to better understand the relationship between the preparation and properties of LPNs. First, additional processing parameters should be investigated to optimize the appearance and texture of LPNs. Second, the molecular interactions of food additives (e.g., alkali, salt, sodium alginate, and guar gum) with other ingredients or starch raw materials require elucidation to clarify the underlying mechanisms. Third, nutritional evaluation (e.g., protein content) should be conducted prior to potential therapeutic application. Fourth, although LPNs possess sensory properties comparable to those of regular noodles, direct comparative studies are necessary, as sensory performance may influence patient compliance. Fifth, incorporating texture and tensile tests into sensory evaluations could help disentangle the influence of human factors, such as measurement variability and psychological bias. Sixth, the effects of varying salt and alkali concentrations on the preparation and properties of LPNs warrant systematic investigation to understand detailed molecular interactions. Seventh, the in vivo digestion of LPNs may differ from in vitro results due to the complexity of the human gastrointestinal tract; thus, in vivo digestion studies should be undertaken to assess potential nutritional effects in patients. Finally, further optimization of the sensory and nutritional properties of LPNs could be achieved by exploring alternative additives or processing techniques.

## Author Contributions

**Cuiping Shi:** investigation. **Wei Cai:** supervision. **Ye Zi:** methodology, data curation, investigation, writing – original draft. **Jian Zhong:** conceptualization, data curation, supervision, funding acquisition, writing – review and editing. **Zhenfeng Liu:** investigation.

## Funding

This research has been supported by a research grant from the Shanghai Municipal Three Year Action Plan for Strengthening the Construction of Public Health System (2023–2025) Discipline Leader Project (GWVI‐11.2‐XD19).

## Conflicts of Interest

The authors declare no conflicts of interest.

## Supporting information


**Figure S1:** Springiness and resilience of the cooked wet noodles and cooked dry noodles with different flours.
**Figure S2:** Textural properties of the doughs with different powders.
**Figure S3:** Cooked wet low‐protein noodles (LPNs) with different designated widths and without alkali/salt addition.
**Figure S4:** Springiness and resilience of the cooked wet noodles and cooked dry noodles with different width and without alkali/salt addition.
**Figure S5:** Springiness and resilience of the cooked dry noodles with different drying temperatures and without alkali/salt addition.
**Figure S6:** Springiness and resilience of the cooked wet noodles with different cooking times and without alkali/salt addition.
**Figure S7:**. Cooked dry and squeezed cooked dry LPNs with different cooking times and without alkali/salt addition.

## Data Availability

The data that support the findings of this study are available from the corresponding author upon reasonable request.
