# Supplementary material for: Synergistic Effects of Alkali, Salt, and Thickness Reduction on the Preparation and Properties of Low‐Protein Noodles
Source: Food Sci Nutr. 2026 May 15;14(5):e71870. doi: 10.1002/fsn3.71870 (PMC13176951; doi:10.1002/fsn3.71870)
Supplement: Supplementary file 1 — Figure S1: Springiness and resilience of the cooked wet noodles and cooked dry noodles with different flours. Figure S2: Textural properties of the doughs with different powders. Figure S3: Cooked wet low‐protein noodles (LPNs) with different designated widths and without alkali/salt addition. Figure S4: Springiness and resilience of the cooked wet noodles and cooked dry noodles with different width and without alkali/salt addition. Figure S5: Springiness and resilience of the cooked dry noodles with different drying temperatures and without alkali/salt addition. Figure S6: Springiness and resilience of the cooked wet noodles with different cooking times and without alkali/salt addition. Figure S7:. Cooked dry and squeezed cooked dry LPNs with different cooking times and without alkali/salt addition. [file FSN3-14-e71870-s001.docx]

**Supplementary Materials:**

**Synergistic Effects of Alkali, Salt, and Thickness Reduction on the** **Preparation and Properties of Low-Protein Noodles**

Ye Zi^1,2,3^, Cuiping Shi^1,2,3^, Zhenfeng Liu^4^, Wei Cai^1,2,3,*^, Jian Zhong^1,2,3,*^

^1^ Shanghai Key Laboratory of Pediatric Gastroenterology and Nutrition, Xinhua Hospital, Shanghai Jiao Tong University School of Medicine, Shanghai 200092, China

^2^ Department of Clinical Nutrition, College of Health Science and Technology, Shanghai Jiao Tong University School of Medicine, Shanghai 200135, China

^3^ Medical Food Laboratory, Shanghai Institute for Pediatric Research, Shanghai 200092, China

^4^ Shanghai Pharma Qingchunbao-Xinhua Hospital Precision Nutrition Research Center, Chiatai Qingchunbao Pharmaceutical Group Limited, Hangzhou 310001, Zhejiang, China

^*^Corresponding authors at:

Shanghai Key Laboratory of Pediatric Gastroenterology and Nutrition, Xinhua Hospital, Shanghai Jiao Tong University School of Medicine, Shanghai 200092, China. E-mail: jzhong@shsmu.edu.cn (J. Zhong), caiw1978@163.com (W. Cai)

Abbreviated running title: Preparation optimization of low-protein noodles

**
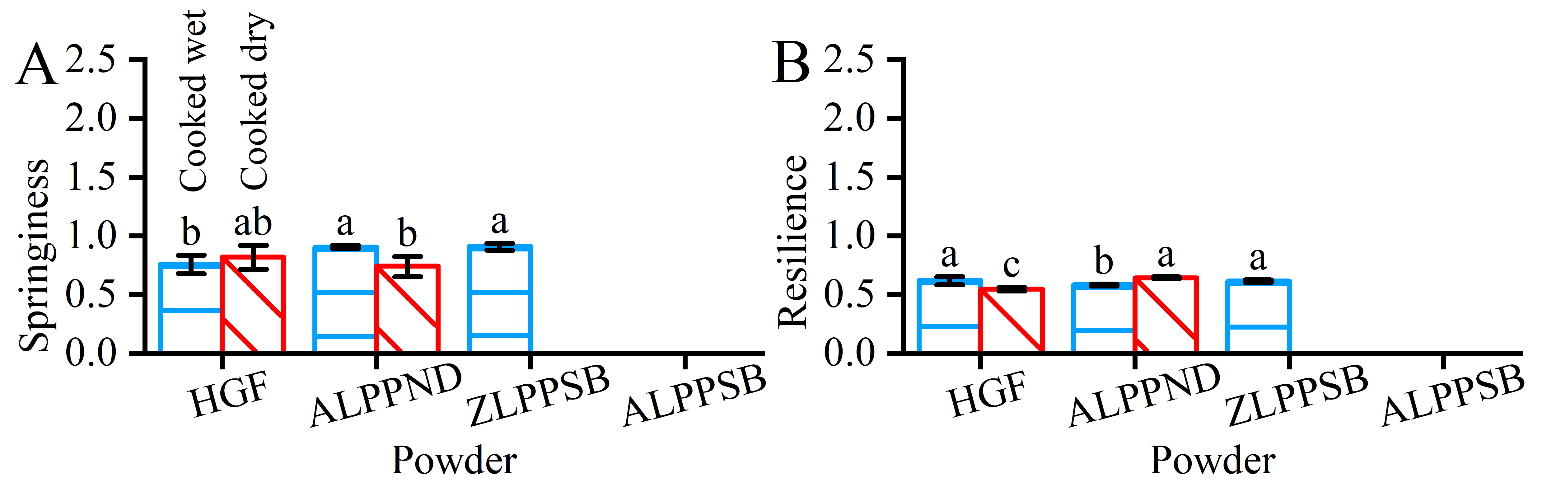
**

**Fig. S1.** Springiness and resilience of the cooked wet noodles and cooked dry noodles with different flours. No alkali and salt were used in the noodles. The powders are high-gluten flour (HGF), ALPPND, Aishushu^TM^ low-protein powders for steamed bun (ALPPSB), and Zhongen^TM^ low-protein powder for steamed bun (ZLPPSB). The preparation conditions were as follows: designated width, 2.5 mm; designated thickness, 1.5 mm; drying temperature, 50°C; cooking time, 8 min. Data are means ± standard deviations (error bars). Different letters on the column indicate significant differences (p < 0.05) in each image.

**
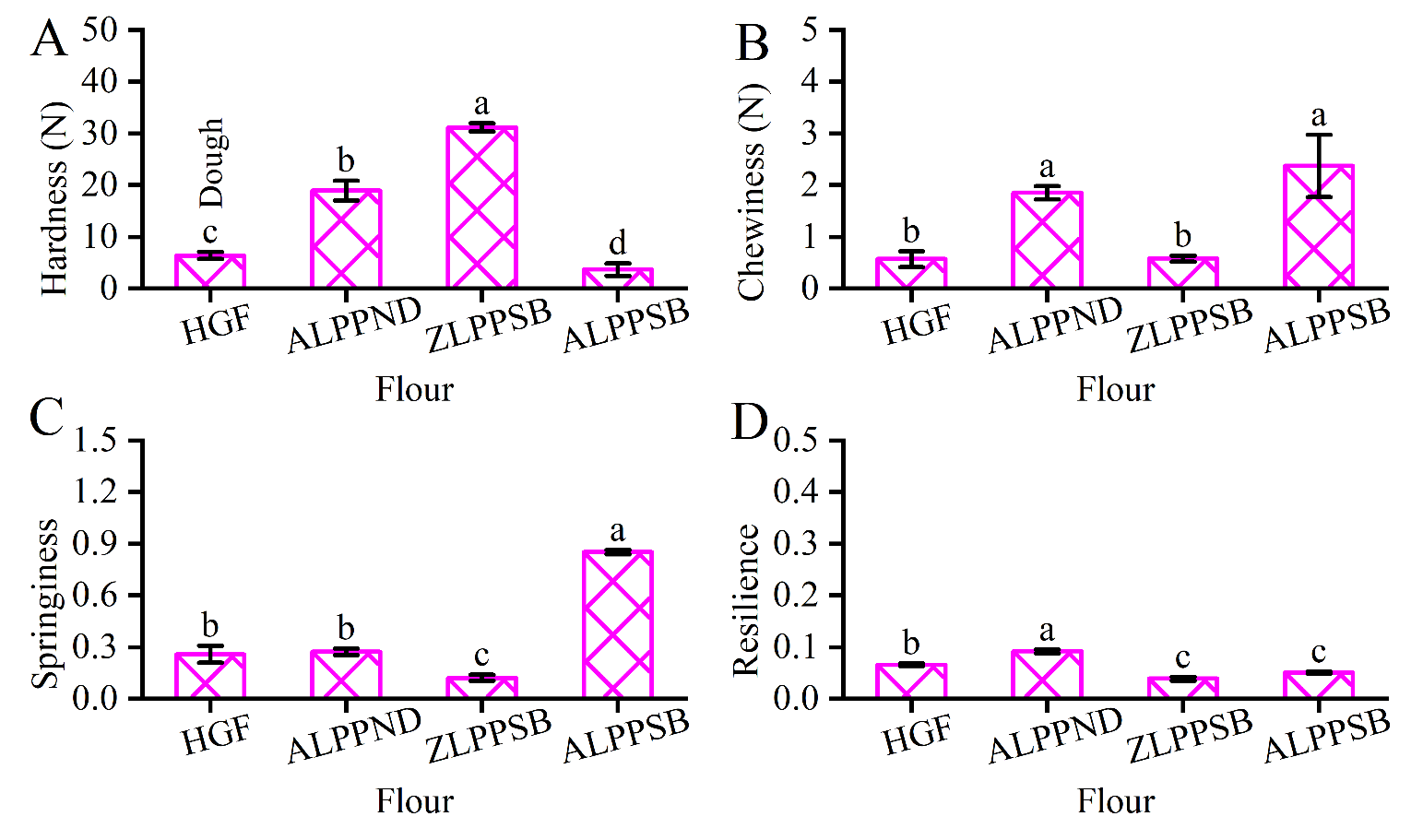
**

**Fig. S2.** Textural properties of the doughs with different powders. No alkali and salt were used in the noodles. The smooth doughs were prepared with different powders with drinking water (52 g/100 g powders). The powders are high-gluten flour (HGF), ALPPND, Aishushu^TM^ low-protein powders for steamed bun (ALPPSB), and Zhongen^TM^ low-protein powder for steamed bun (ZLPPSB). Data are means ± standard deviations (error bars). Different letters on the column indicate significant differences (p < 0.05) in each image.

**
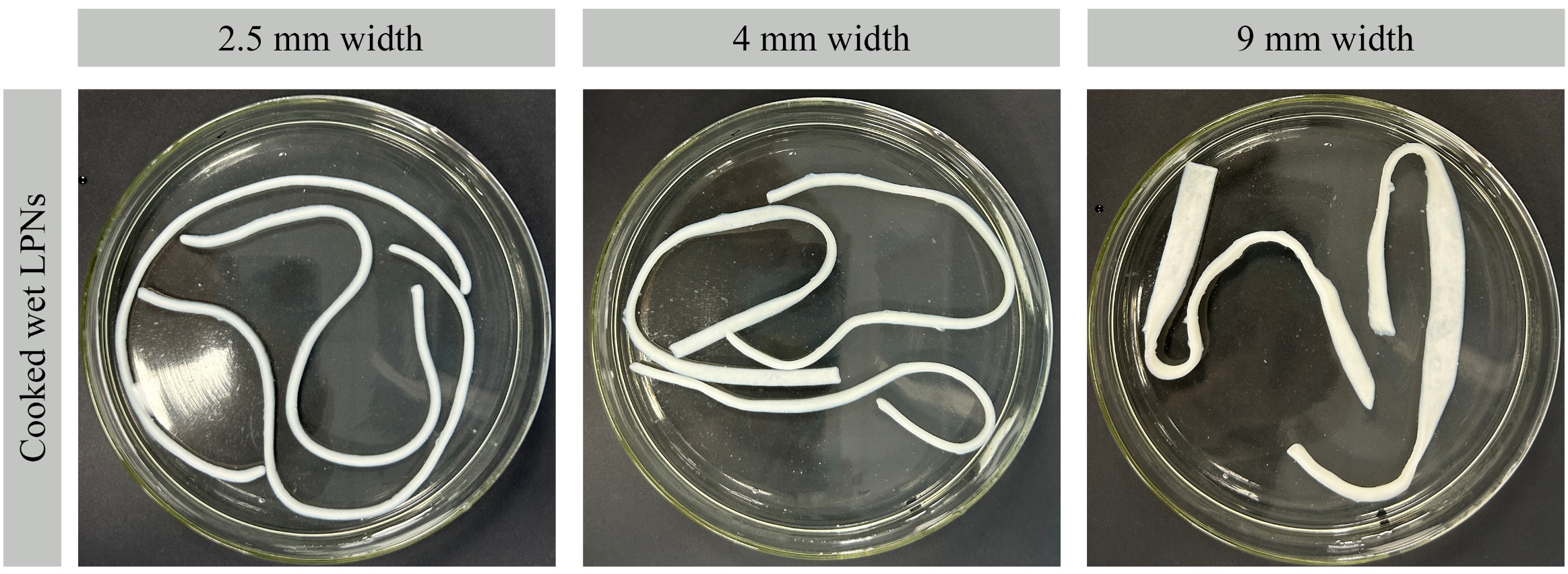
**

**Fig. S3:** Cooked wet low-protein noodles (LPNs) with different designated widths and without alkali/salt addition. The other preparation conditions were as follows: Powder, ALPPND; designated thickness, 1.5 mm; drying temperature, 50°C; cooking time, 8 min.


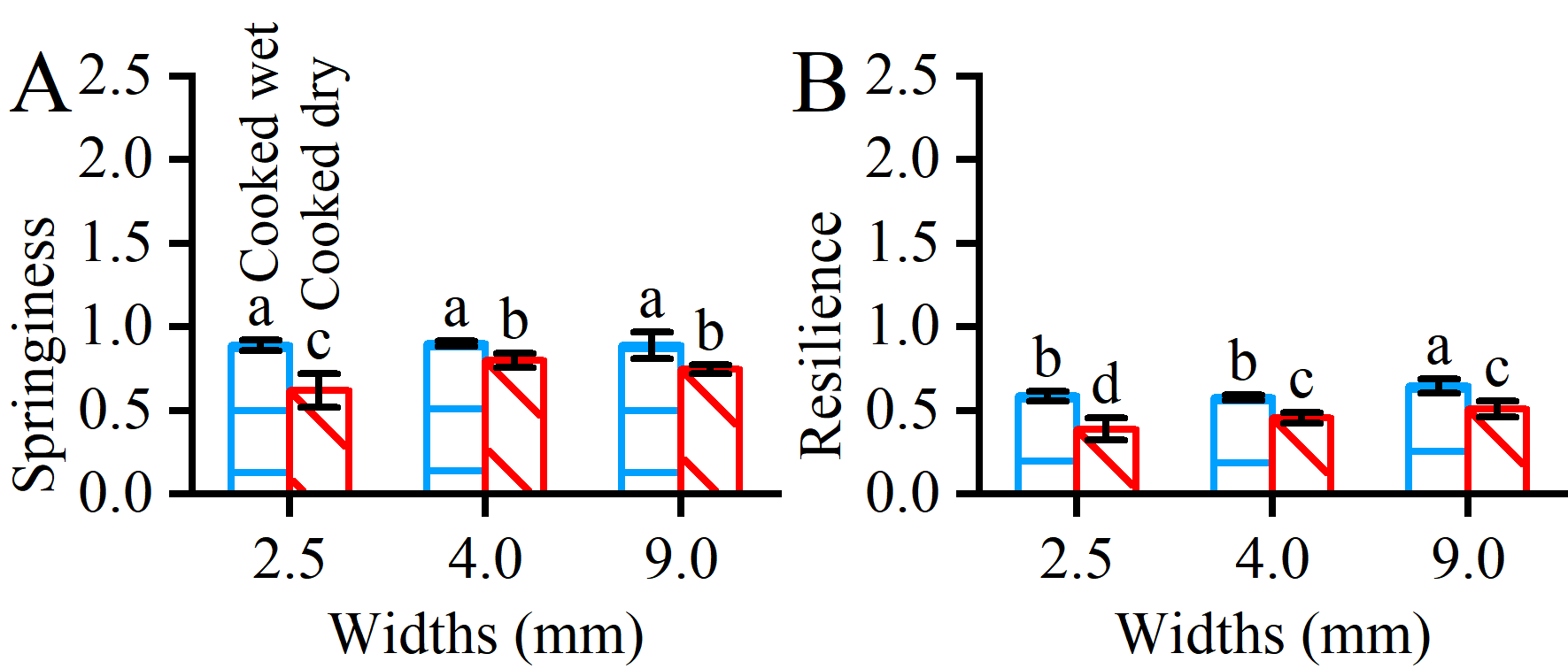


**Fig. S4.** Springiness and resilience of the cooked wet noodles and cooked dry noodles with different width and without alkali/salt addition. The other preparation conditions were as follows: Powder, ALPPND; designated thickness, 1.5 mm; drying temperature, 50°C; cooking time, 8 min. Data are means ± standard deviations (error bars). Different letters on the column indicate significant differences (p < 0.05) in each image.


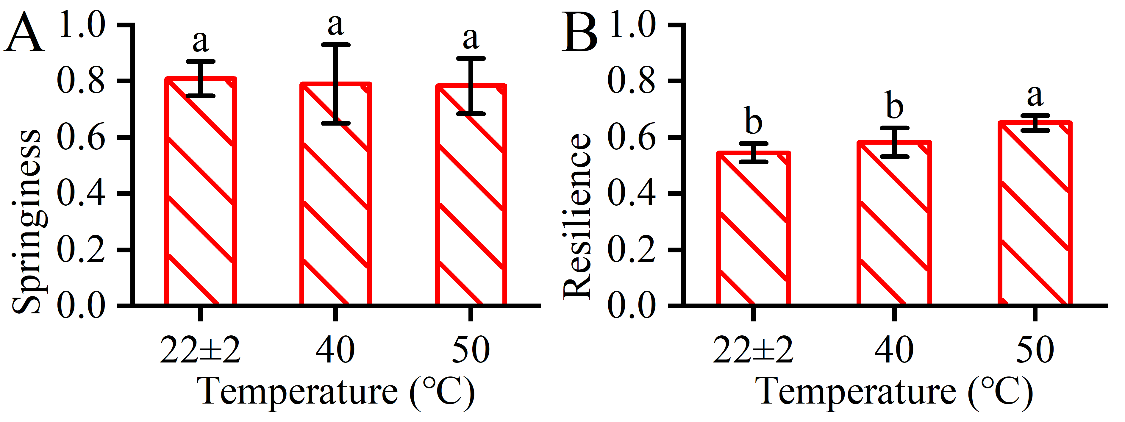


**Fig. S5.** Springiness and resilience of the cooked dry noodles with different drying temperatures and without alkali/salt addition. The other preparation conditions were as follows: Powder, ALPPND; designated width, 2.5 mm; designated thickness, 1.5 mm; cooking time, 8 min. Data are means ± standard deviations (error bars). Different letters on the column indicate significant differences (p < 0.05) in each image.


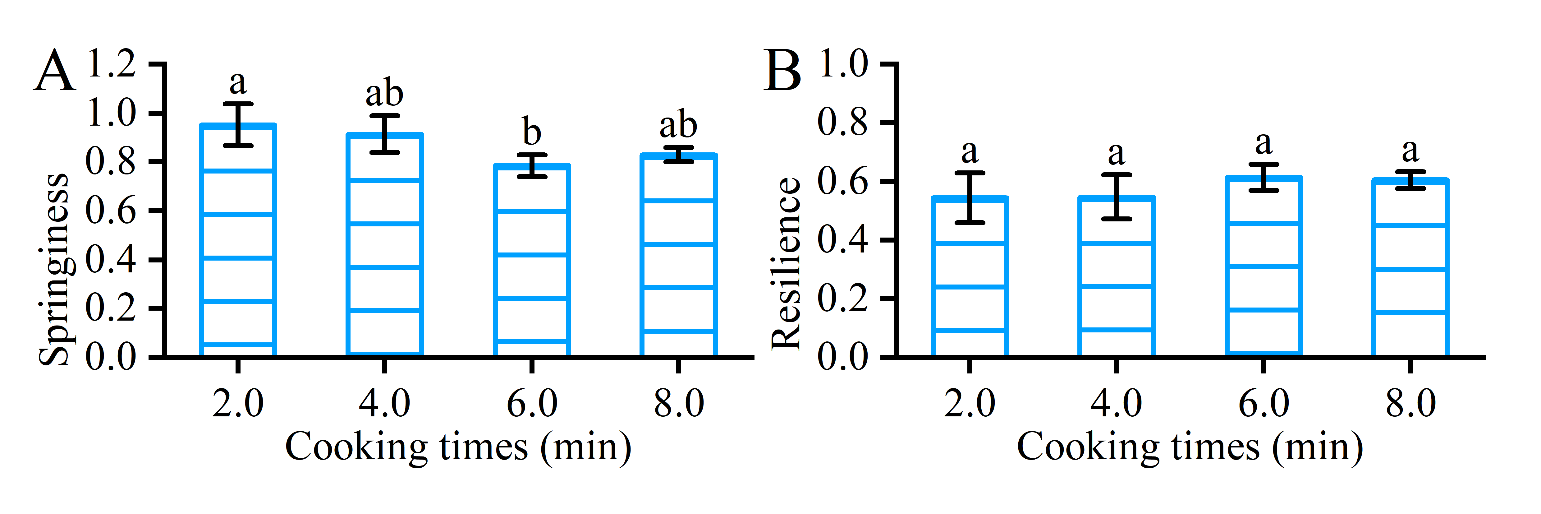


**Fig. S6.** Springiness and resilience of the cooked wet noodles with different cooking times and without alkali/salt addition. The other preparation conditions were as follows: powder, ALPPND; designated width, 2.5 mm; designated thickness, 1.5 mm; drying temperature, 50°C. Data are means ± standard deviations (error bars). Different letters on the column indicate significant differences (p < 0.05) in each image.

**
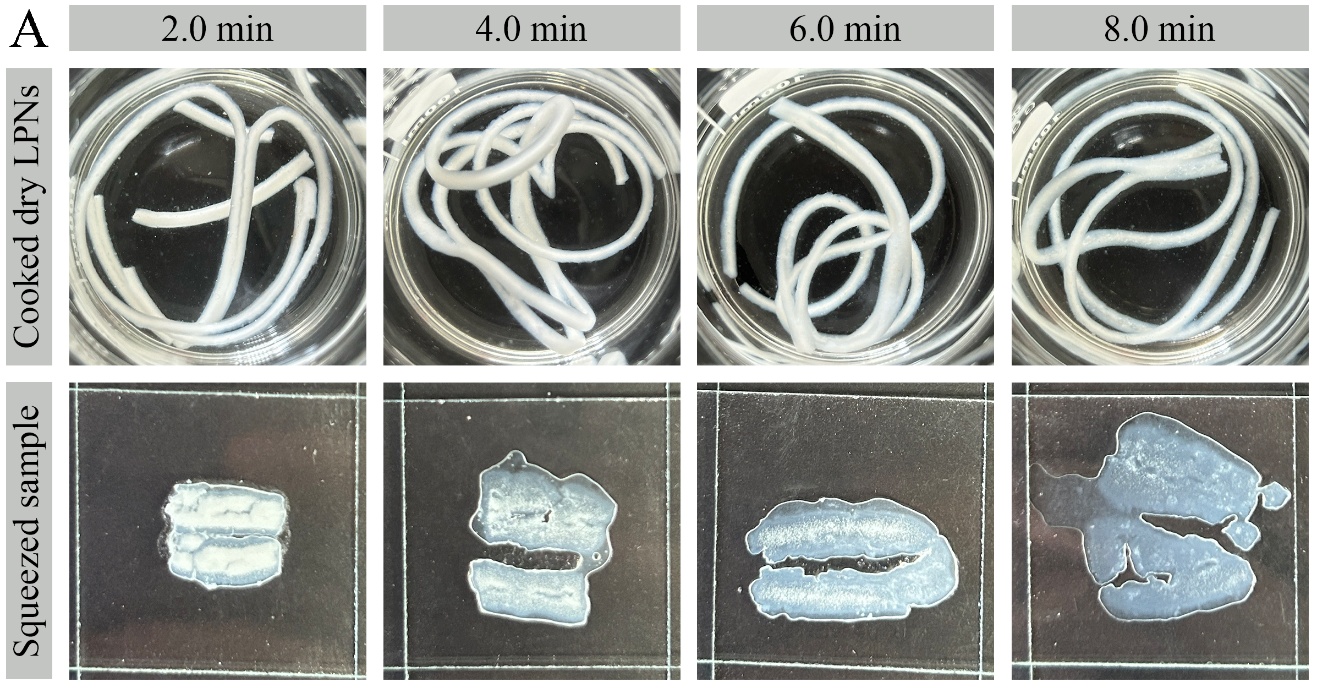
**

**Fig. S7**. Cooked dry and squeezed cooked dry LPNs with different cooking times and without alkali/salt addition. The other preparation conditions were as follows: powder, ALPPND; designated width, 2.5 mm; designated thickness, 1.5 mm; drying temperature, 50°C.
